# Clinical and Molecular Characterization of a Patient with Generalized Arterial Calcification of Infancy Caused by Rare *ABCC6* Mutation

**DOI:** 10.3390/jpm14010054

**Published:** 2023-12-30

**Authors:** Ruen Yao, Fan Yang, Qianwen Zhang, Tingting Yu, Ying Yu, Guoying Chang, Xiumin Wang

**Affiliations:** 1 Department of Medical Genetics and Antenatal Diagnostic Center, Hainan Branch, Shanghai Children’s Medical Center, School of Medicine, Shanghai Jiao Tong University, Sanya 572022, China; yaoruen@scmc.com.cn (R.Y.);; 2 Department of Medical Genetics and Molecular Diagnostic Laboratory, Shanghai Children’s Medical Center, School of Medicine, Shanghai Jiao Tong University, Shanghai 200127, China; 3 Clinical Research Ward, Shanghai Children’s Medical Center, School of Medicine, Shanghai Jiao Tong University, Shanghai 200127, China; yangfan@scmc.com.cn (F.Y.);; 4 Department of Endocrinology and Metabolism, Shanghai Children’s Medical Center, School of Medicine, Shanghai Jiao Tong University, Shanghai 200127, China

**Keywords:** *ABCC6* gene, generalized arterial calcification, exon deletion, genetic diagnosis, pseudoxanthoma elasticum

## Abstract

Generalized arterial calcification of infancy (GACI) is a rare autosomal-recessive disease characterized by extensive arterial calcification in infancy, with clinical manifestations such as arterial stenoses and heart failure. The ENPP1 inactivation mutation has been identified as a potential defect in most of the cases of GACI, while mutations in *ABCC6* are demonstrated in patients who are genotyped as pseudoxanthoma elasticum and only limited cases of GACI are reported. Whole-exome sequencing was applied for the detection of pathogenic variants. Copy-number variants of pathogenic genes were also evaluated through a bioinformatic process and were further validated by real-time quantitative PCR. In this report, we described the clinical information and treatment of a patient with extensive arterial calcification. We have identified the underlying cause as biallelic mutations in *ABCC6* (NM_00117: exon30, c.4223_4227dupAGCTC p.(Leu1410Serfs*56)) and a unique exonic deletion that spans from the first to the fourth exons of *ABCC6* (chr16:16313388-16330869)). This discovery was made by utilizing a combined genetic testing approach. With the review of previously reported GACI patients with *ABCC6* mutation, our work contributed to enriching the mutation spectrum of GACI and providing further information on this rare form of inherited disorder.

## 1. Introduction

Generalized arterial calcification of infancy (GACI; OMIM 208000) is a rare but life-threatening disease that was first described in 1899, which is characterized by muscular arterial fibrosis, endosomal hyperplasia, calcification of the inner elastic membrane, and resulting arterial stenosis [1]. The affected patients had severe myocardial ischemia, congestive heart failure, edema, cyanosis, respiratory distress, cardiomegaly, and hypertension. The results of the radiologic examination indicated the presence of widespread calcification in the soft tissues surrounding the blood vessels and joints. It is important to note that some patients may experience a milder form of hypophosphate rickets as a result of their condition [2]. GACI is estimated to affect about 1 in 200,000 pregnancies, with a carrying rate of approximately 1 in 200 [1]. Survival rates vary widely, but mortality is high, and most patients (60%) die within the first six months of life. Additionally, a significant percentage of 24.4% pass away while still in the uterus or are stillborn [3,4]. It is worth noting that congestive heart failure, myocardial infarction, persistent arterial hypertension, and multiple organ failure are responsible for a significant number of deaths. In some rare cases, there may be spontaneous regression with age, while those who survive into adulthood may experience musculoskeletal complications, enthesis mineralization, and cervical spine fusion.

Just to clarify, GACI is caused by biallelic inactivated variants in hexanucleotide pyrophosphatase/phosphodiesterase 1 (ENPP 1; OMIM 173335) in 75% of cases [5]. On the cell surface, there is an enzyme encoded by ENPP 1 that has the ability to break down ATP into AMP and inorganic pyrophosphate (PPi). When PPi is lacking, it can lead to vascular calcification since PPi is the primary inhibitor of physiological calcification. In around 9–10% of GACI cases, a specific genetic mutation has been identified in a gene called *ABCC6* (OMIM 603234). This gene plays a crucial role in the cellular ATP export process and produces a plasma membrane transporter that is predominantly found in the liver. Pseudoxanthoma elastica (PXE; OMIM 264800) is a condition that results from a genetic mutation in *ABCC6*, which causes calcification and breakage of elastic fibers. This condition primarily affects the skin, leading to pimples in the flexion and neck, but can also impact the cardiovascular system, causing adult onset arterial calcification, and the retina, resulting in orange, vascular streaks, and choroidal neovascularization [6]. It is not clear which molecule of *ABCC6* is transported to the extracellular space, but ATP is a possible candidate. ATP is a substrate for ENPP 1 and a source of plasma PPi. When *ABCC6* is deficient, PPi levels decrease in both animal models and humans. When the *ABCC6* gene is defective, it causes calcium to accumulate in the arterial wall, which, in turn, leads to arterial calcification and narrowing due to intimal proliferation [7]. It has been difficult to establish a definitive agreement on the clinical diagnostic standards for systemic arterial calcification in infants. This is due to the fact that there is an overlap in appearance between GACI and PXE, which complicates matters [8].

In this report, we discuss a six-year-old girl who was diagnosed at the age of five, outlining the details of her case. She presented with extensive arterial calcification (including the aorta, heart valves, intracranial, both kidneys, pancreas, spleen, adrenal glands, mesentery, and lymph node) and hypertension. A comprehensive genetic testing strategy including whole exome sequencing, copy number evaluation, and real-time quantitative PCR (q-PCR) revealed the genetic pathogenesis. Compound heterozygous mutation in the *ABCC6* gene including a maternally inherited frameshift variant (c.4223_4227dupAGCTC p.L1410Sfs*56) and a paternally inherited exon deletion of the gene. Detailed clinical and genetic evaluation of our case and review of reported *ABCC6*-related GACI cases contributed to the mutation spectrum and geno-phenotypic relationship of GACI caused by *ABCC6* gene variations

## 2. Methods

### 2.1. Whole Exome Sequencing and Copy Number Evaluation

Peripheral blood samples were collected from the proband and parents. The sequencing library was constructed using the SureSelect Human All Exon V6 enrichment kit (Agilent, Santa Clara, CA, USA). The library preparation experimental process included the enzymatic digestion of DNA samples, library hybridization, and library amplification and purification. The Nova6000 sequencing platform (Illumina, Inc., San Diego, CA, USA) was used for the high-throughput sequencing. FastQC version 0.11.9 (Babraham Research Institute, Cambridge, UK) and Fastp version.0.20.1 (Visible Ge-netics, Inc., Toronto, ON, Canada) was used for data quality control and to remove the adaptor sequence. SpeedSeq version 0.1.2 (Ira Hall Lab, St. Louis, MO, USA) was applied for sequencing read alignment to the reference genome (GRCh37/hg19). Further, bamdst (version 1.0.9) and mosdepth (version 0.3.1) were used to count sequencing in-dexes of BAM files after alignment, including average sequencing depth, coverage rate, mapping rate, and polymerase chain reaction duplication rate. Genome Analysis Toolkit version (GATK) 4.2.0.0 (Broad Institute, Cambridge, MA, USA) was used to detect variants in the BAM file, passing a quality control test that was performed following the best practice guidelines. Genome-wide copy number variants (CNVs) were identified with the CNVkit software (version v0.9.10), which is a tool kit that can infer and visualize copy number states from targeted DNA sequencing data and whole genome data.

### 2.2. Real-Time Quantitative PCR

Exon copy numbers of *ABCC6* were determined by real-time quantitative PCR detection using the DNA-binding dye SYBR Green I. *GAPDH* was introduced simultaneously in the system as a reference gene to avoid variants related to DNA input amount or the presence of a PCR inhibitor. The method involves amplifications of the first three exons of *ABCC6* gene and one exon of *GAPDH*. All primer sequences for RT-PCR were designed using Primer 3, and detailed sequence information was listed in Table 1. The SYBR Green I amplification mixtures (15 mL) were composed of SYBR Green I master mix (Eurogentec, Seraing, Belgium), 250 nM each of forward and reverse primers, 10 nM fluorescein, and 10 ng template DNA. Cycling conditions comprised an initial denaturation step of 10 min at 95 °C, followed by 40 cycles at 95 °C for 15 s and 60 °C for 60 s. After PCR amplification, a melting curve was generated for each PCR product to assess the specificity of the reaction. Gene copy number calculation utilized the comparative (delta-Ct) Ct method.

## 3. Results

### 3.1. Clinical Information

**Figure 1:**
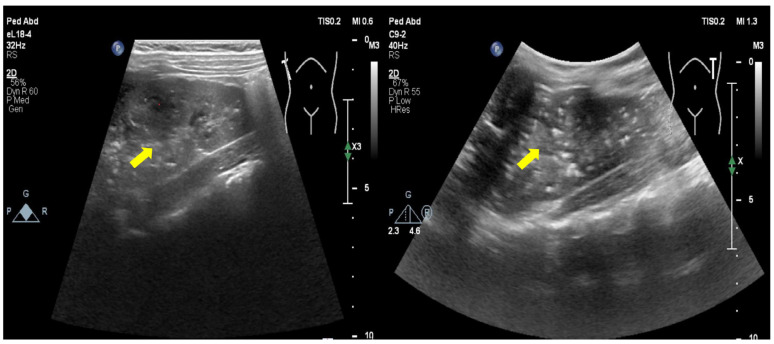
Ultrasound showed scattered calcifications in both kidneys.

The proband is the only child born to a non-consanguineous couple of Chinese ancestry with no notable family history. She was born at term following an uneventful pregnancy. Her growth and development were normal, and she had frequent abdominal pain since she was one year old without receiving specific treatment. She has a history of heel fractures and becomes tired easily. When she was 5 years old, she went to the hospital because of “chest pain and dyspnea”. Examination revealed high blood pressure (120–140/80–100 mmHg) and calcification in multiple parts of her body, including the aorta, heart valves, intracranial, both kidneys, pancreas, spleen, adrenal glands, mesentery and lymph nodes (Figure 1 shows the ultrasound image of the kidney). Fundus examination showed detachment of retinal pigment epithelium. Liver and kidney function, blood lipids, blood coagulation, myocardial enzymes, inflammation, immunity, infection indicators, thyroid function, and hearing screening were normal. The bone age matched the actual age, and the ultrasound of the thyroid and parathyroid glands revealed no irregularities. Bone metabolism related: PTH 154.6↑ −48.0 pg/mL (reference values: 15–65 pg/mL), blood Ca 2.40 mmol/L (2.13–2.7 mmol/L), P 1.76 mmol/L (1.45–2.10 mmol/L), ALP 323U/L (42–390 U/L), VitD 41.40 nmol/L (≥30 ng/mL). She took creatine phosphate and blood pressure-lowering drugs. The patient began taking metoprolol and amlodipine besylate for hypertension when they were 5 years and 2 months old. These medications effectively managed their blood pressure. The child was treated with zoledronic acid injection. The systemic vascular calcification did not improve significantly, but abnormal calcium and phosphorus metabolism was corrected after four doses (each dose was 0.0125 mg/kg at an interval of 2 months).

### 3.2. Genetic Diagnosis Based on Whole Exome Sequencing

**Figure 2:**
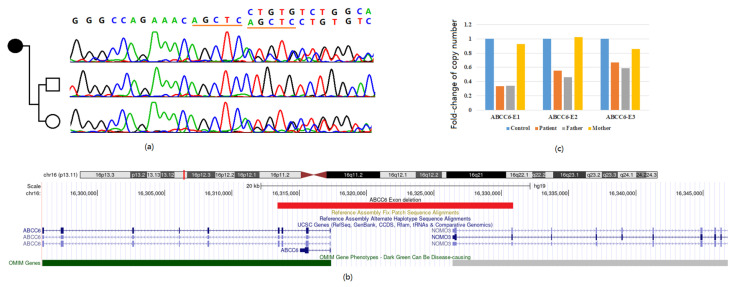
(**a**) Sanger sequencing revealed a frameshift variant inherited from the mother; (**b**) Sketch diagram of exon deletion range of the proband prompted by sequencing depth information; (**c**) Exon deletion confirmation by real-time quantitative-PCR.

Whole-genome sequencing revealed a heterozygous *ABCC6* mutation NM_00117: exon30, c.4223_4227dupAGCTC (p.Leu1410Serfs*56) (Figure 2a). This variant could be easily classified as pathogenic (PVS1 + PM2 + PP4). Sanger sequencing confirmed the mother as an asymptomatic carrier of the variants. Further evaluation of copy number variations based on read-depth information of original sequencing data revealed an exonic deletion encompassing the first to the fourth exons of *ABCC6* and the first two exons of *NOMO3* with minimum interval chr16:16313388–16330869. (Figure 2b).

### 3.3. Confirmation of the Exon Deletion

Due to the existence of a pseudogene of *ABCC6* in the human genome, confirmation of exon deletion was carried out in three exons of *ABCC6* with successfully designed q-PRC primers. Compared with the control sample and reference gene signal, the proband and his father showed a significant fold-change decrease in the first three exons of *ABCC6*, consistent with deletion detection from the exome sequencing result (Figure 2c).

### 3.4. Reported Cases and Literature Review

An analysis of the international GACI registry found that 9 out of 15 patients had biallelic *ABCC6* mutations and 6 out of 15 had monoallelic *ABCC6* mutations, which includes the case being discussed [7,9]. Vascular calcification is the earliest and most prominent feature of GACI. All of the patients were presented with typical symptoms of severe infantile arteriopathy. It has been reported that the connection between *ABCC6* mutations and hypophosphatemia is not very specific. There has only been one case of a hypophosphatemic rickets in a patient with GACI who had a monoallelic *ABCC6* mutation. Additionally, early-onset hearing loss may be linked to GACI caused by ENPP1 mutations, and this connection appears to be multifactorial [10]. It is worth noting that so far, there have not been any reports of hearing loss in patients with *ABCC6* mutations that lead to GACI. Medical researchers have found that infants who have mutations in the *ABCC6* gene can develop a serious condition of systemic arterial calcification. Tragically, out of a group of 15 infants affected by this disease, 5 have succumbed to it within the first year of their life (Table 2). It has been observed that amino acid exchanges resulting from mutations occurred in the cytosolic or transmembrane domains of the *ABCC6* protein. These regions are believed to play a crucial role in the protein’s function. You can see the localization of these mutations in Figure S1.

## 4. Discussion

GACI is a genetic disorder that affects the overall functioning of the circulatory system and is considered rare. The most severe cause of morbidity and mortality in patients with GACI is vascular calcification. Medium and large-diameter arteries may exhibit severe calcification, rupture of elastic fibers, calcification of the inner elastic layer, and the proliferation of smooth muscle cells in the intimal layer [5]. Arterial calcification and narrowing can be severe and can occur during the fetal development stage, with the earliest signs of calcification being visible at 18 weeks. Alternatively, it can also happen in the weeks following the birth of a child [11]. Therefore, in cases of survival after infancy, vascular involvement may affect organs such as the kidneys, liver, and spleen. GACI usually occurs during fetal development, thus sometimes diagnosed before birth, but more often diagnosed after birth [9]. Decreased levels of pyrophosphate in the blood, leading to increased calcification, narrowing vessels and arteries, and restricting blood flow is the core pathogenesis of GACI.

Artery and vessel abnormality in GACI patients can lead to several health problems like hypertension, heart disease, and kidney disease at birth. It can also increase the risk of stroke, heart attack, and kidney failure [11]. Postmortem studies have revealed that the location of calcified arteries in GACI varies depending on the time of disease onset [11]. Arterial calcification is a common occurrence in individuals with GACI. In early-onset GACI, the hepatic and aortic arteries are the most commonly affected (with a prevalence of ≥80%), followed by the coronary, pulmonary, and renal arteries. On the other hand, in late-onset GACI, the coronary artery is the most commonly affected (with a prevalence of 88%), followed by the renal, pulmonary, aorta, adrenal, splenic, pancreatic, and mesenteric arteries. In the case of long-term GACI survivors, arterial calcification is most commonly observed in the aorta, renal artery, mesenteric artery, coronary artery, iliac artery, and pulmonary artery [1]. Individuals who do not show signs of progressive calcification may still have arterial stenosis with intimal thickening, which could be the underlying cause of recurrent miscarriage [1]. Individuals with GACI frequently experience ectopic calcification, with the condition being present in up to 94% of patients. The heart valves are the most commonly affected area, with the kidneys following closely behind [7,9]. About 30–50% of infants with GACI develop joint calcification, most commonly in the shoulder, hip, ankle, wrist, and sternoclavicular joints [1]. Other extravascular calcifications include brain parenchyma, earlobes, myocardium, ligaments, and Achilles tendons [1,12].

It is important to mention that around half of GACI cases (48%) are identified before birth, while the other half (52%) are usually diagnosed when the individual is approximately 3 months old [11]. In our statistical summary, we found that the median age of diagnosis was 2 months. The diagnosis of GACI disease can be complex and may involve a combination of clinical observations, histopathological findings, imaging, and genetic testing. The symptoms of GACI disease affecting the cardiovascular system are varied and can include hyper-amniotic fluid, fetal edema, fetal distress, cardiac hypertrophy, hypertension, heart failure, visceral effusion, cyanosis, peripheral pulse reduction, and dyspnea [11]. It is possible to identify blood vessel calcification through prenatal ultrasound. However, calcification can be easily missed during routine radiography, even though it appears as a faint radio-opaque area. This oversight can cause delays in diagnosis. As a result, the preferred imaging technique for evaluating the development of systemic calcification in GACI is whole-body computed tomography.

GACI is a condition that is caused by a genetic variant inherited in a recessive manner. The majority of GACI patients have a variant in the ENPP1 gene, which produces an important enzyme for pyrophosphate generation. Sadly, most infants with GACI and ENPP1 variants usually experience arterial and vessel hardening symptoms and often do not survive beyond the first six months of life [13]. GACI can also result from mutations in the *ABCC6* gene, which produces the MRP6 protein. *ABCC6* proteins carry molecules across cell membranes, although MRP6’s transported substances remain largely unknown. Jansen et al. found that the absence of *ABCC6* reduces the amount of extracellular adenosine triphosphate (ATP), a vital substrate necessary for extracellular PPi production [14]. It has been observed through various studies that *ABCC6* aids in the release of adenosine triphosphate (ATP) into the extracellular space, although the mechanism behind this is still unknown. Once released, ATP is quickly broken down into adenosine monophosphate (AMP) and pyrophosphate. The presence of pyrophosphate hinders mineral deposition (calcification) [15]. When *ABCC6* is absent, it can cause a decrease in serum levels of pyrophosphate. This is due to the downregulation of ENPP1 and NT5E gene expression, which leads to a reduction in plasma levels of PPi. As a result of lowered adenosine production, TNAP can become activated [16,17]. A study conducted on a limited number of *ABCC6* mutants has revealed two possible molecular consequences of these mutations. The first one is transport deficiency, which results from the failure to hydrolyze ATP, while the second one is abnormal protein folding that leads to reduced trafficking and/or intracellular retention [6]. *ABCC6* is predominantly present in the liver and kidneys, although it is also found in trace amounts in various other tissues such as the skin, stomach, blood vessels, and eyes [18]. Individuals with GACI caused by *ABCC6* deficiency age may exhibit symptoms similar to those of PXE, which is another autosomal recessive disorder that is also associated with mutations in the *ABCC6* gene [19].

*ABCC6* variant carrier individuals typically present with PXE, a condition where calcifications usually appear in older children and adults, as opposed to GACI [19]. Individuals with GACI minor ENPP1 deficiency have been found to exhibit ocular and cutaneous characteristics that are similar to those typically observed in PXE [20]. Further investigation is required to explain why some patients with *ABCC6* mutations experience the severe GACI phenotype, which can lead to myocardial infarction and death in early infancy, while others experience a relatively mild phenotype of PXE. One possibility is that other genes that regulate artery calcification may be involved. Therefore, it may be beneficial to test GACI patients who carry *ABCC6* mutations for mutations in genes that encode other inhibitors of artery calcification [12]. The overlap between genotype and phenotype suggests that mutations in *ABCC6* and ENPP1 could affect similar physiological processes. This indicates that GACI is an extreme and severe end of the vascular phenotype spectrum of PXE. However, it is important to note that a single heterozygous pathogenic variant in both ENPP1 and *ABCC6* is not enough to cause GACI, as per a reported pedigree [21]. The pathophysiological relation between mutation in both genes and clinical consequences as GACI or PXE need further confirmation. At the same time, GACI patients shared almost similar mutation spectrum in the *ABCC6* gene with PXE patients [22]. The reason for divergent phenotypes resulting from the same mutation remains unclear. Genetic modifier genes or epigenetic factors were suspected in these situations. On the other hand, diet, lifestyle variables, and environmental factors could also act as modifiers for phenotypic presentations of PXE patients [23]. The clinical diagnosis of GACI is relatively direct and could guide the direction of genetic diagnosis. However, six patients were diagnosed with GACI but with only the monoallelic *ABCC6* variant [7]. The existence of another allelic disorder, like exon deletion in our case or other cryptic variants, is worthy of investigation.

At the moment, there are no permanent solutions for PXE and GACI. The treatments available are merely intended to alleviate certain symptoms [12,24]. To ensure effective treatment for ectopic arterial calcification, it is essential to monitor systemic diseases affecting various organs and provide personalized care for each organ. This requires the collaboration of an integrated medical team comprising multiple management specialties. Additionally, it is crucial to start treatment interventions as early as possible since low PPi levels can lead to diffuse arterial calcification, which is associated with high mortality rates in the prenatal and early infancy stages [25]. Researchers have conducted numerous studies to understand the process of calcification, leading to the development of innovative treatment methods for PXE and GACI. Bisphosphonates, which are non-hydrolyzable pyrophosphate (PPi) analogs, possess properties that can block enzymes that utilize pyrophosphate. These compounds have been used for several years in treating osteoporosis, Paget’s disease of bone, and other mineralization-related applications [3]. It has been observed that the use of bisphosphonate treatment can lower the mortality rate of individuals with GACI by 65% [12]. Bisphosphonates are commonly used to treat GACI patients due to their effectiveness. These drugs contain a non-hydrolyzed carbon P-C-P motif at their core, which produces more stable PPi analogs. Bisphosphonates come in two types: nitrogen-containing and nitrogen-free compounds. The first generation of nitrogen-free bisphosphonates, such as etidronate disodium, stops bone mineralization by binding to hydroxyapatite. Meanwhile, subsequent generations of nitrogen-containing bisphosphonates, such as pamidronate disodium, are more effective in inhibiting the mevalonate pathway and have superior anti-absorption properties. Both types of bisphosphonates lead to osteoclast apoptosis by interfering with proteins that regulate bone metabolism. However, the effectiveness of bisphosphonates in treating GACI is still subject to debate. A retrospective review of 55 infants with systemic arterial calcification showed that bisphosphonate therapy effectively reduced calcification and improved mortality [26]. However, a recent report showed that bisphosphonate treatment did not improve survival. Further research is needed to determine its effectiveness [24]. GACI in infants leads to a decrease in the amount of inorganic pyrophosphate (PPi) present outside the cells, which acts as an inhibitor for the formation of hydroxyapatite [27]. In studies of GACI mice showing ectopic arterial calcification, oral PPi significantly inhibited calcification and reduced calcium load by 75% to 88% [28]. Studies have shown that oral PPi can effectively prevent calcification in the offspring of GACI pregnant mice. However, more research is needed to determine whether oral PPIs are effective in treating GACI in humans. Nevertheless, the U.S. Food and Drug Administration has confirmed the safety of oral PPIs for human use.

According to research, administering soluble recombinant human ENPP 1-Fc protein can prevent myocardial infarction and aortic calcification in mouse models that have ENPP 1 deficiency. This protein increases extracellular PPi levels, which reduces ectopic calcification and impedes the proliferation of vascular smooth muscle cells by cutting extracellular ATP to block AMP and adenosine anti-proliferation signaling [16,25,27].

GACI offers a range of treatment options, including the possibility of a heart transplant. There have been successful cases where children as young as 18 months, suffering from severe myocardial infarction and end-stage heart failure due to diffuse coronary artery calcification, have undergone heart transplants and showed no recurrence of calcification for up to two years [29]. To manage GACI, standard anti-hypertensive therapy with aspirin can be used for individuals with severe coronary stenosis. Additionally, intravitreal VEGF inhibitors can help with choroidal neo-vascularization, while calcitriol oral phosphate supplements are used for hypophosphatemic rickets. Those with hearing difficulties may also benefit from using hearing aids [14]. It is important to note that none of the treatments tried were able to completely clear or reverse pre-existing calcifications in patients suffering from PXE and GACI. Due to the complexity of these conditions, a single treatment method may not be enough, and multiple forms of treatment may be necessary. The patient, in this case, reported that they were satisfied with blood pressure control achieved through a combination of metoprolol and amlodipine besylate. While zoledronic acid did not succeed in reversing systemic vascular calcification, it was able to correct calcium and phosphorus metabolism abnormalities. As systemic calcifications occur due to long-term mineral accumulation, further follow-up is needed to observe the effectiveness of these treatments.

## 5. Conclusions

In conclusion, our clinically diagnosed GACI patient exhibited biallelic *ABCC6* variants, with an additional report of an exon deletion in *ABCC6*-related GACI. Treatment interventions, including hypertension management and zoledronic acid, demonstrated limited improvement in vascular calcification but successful correction of calcium and phosphorus metabolism. The genetic and phenotypic insights from this case, combined with a thorough literature review, contribute to a deeper understanding of GACI and provide valuable treatment considerations.

## Figures and Tables

**Table 1:** Real-time quantitative PCR primer sequences for *ABCC6* exon deletion.

Primers	Sequence	Product Size
*ABCC6*-E1F	5′ TGCTGGGTCCAAAGTGTTTA 3′	469 bp
*ABCC6*-E1R	5′ CAGCCCGAGAGATCTGCAGC 3′	
*ABCC6*-E2F	5′ GATCCAAAAAGTTGCCTGGC 3′	328 bp
*ABCC6*-E2R	5′ TGTCCCCTGCCTCCCCCGAA 3′	
*ABCC6*-E3F	5′ CGCCTACCAGTTTGCTGTGA 3′	221 bp
*ABCC6*-E3R	5′ AAGCCGGGCTCCAGACTGAA 3′	
*GAPDH*-F	5′ CCCCTTCATACCCTCACGTA 3′	192 bp
*GAPDH*-R	5′ ACACCATCCTAGTTGCCTCC 3′	

**Table 2:** Clinical and Mutational Data of Patients Who Have a GACI Phenotype and Carry Mutations in *ABCC6*.

Parameter	Previous Study	This Study
	*ABCC6* Data Set (n = 14) ^a^	
Alive, n (%)	9 (64.3)	1
Age at data collection (months), median (range)	96.9 (2–372)	60
Deceased, n (%)	5 (35.7)	0
Age at death (months), median (range)	2.6 (1.4–5)	0
Gender, female/male (% female)	5/9 (36)	female
Mutation type, biallelic/monoallelic (% biallelic)	8/6 (57.1)	biallelic
Rickets		
Yes/no (% yes of assessed)	1/13 (7.1)	0
Age at diagnosis (months), median (range)	2 (2)	0
Bisphosphonate treatment		
Yes/no (% yes of assessed)	3/11 (21.4)	1/0

^a^ Based on available dates.

## Data Availability

The sequencing raw data are available from the corresponding author on reasonable request.

## Supplementary Materials

The following supporting information can be downloaded at: https://www.mdpi.com/article/10.3390/jpm14010054/s1, Figure S1: Summary of *ABCC6* gene mutations that cause GCAI disease, including this study (red text) and literature (black text).
