# Supplementary material for: Clinical and Molecular Characterization of a Patient with Generalized Arterial Calcification of Infancy Caused by Rare ABCC6 Mutation
Source: J Pers Med. 2023 Dec 30;14(1):54. doi: 10.3390/jpm14010054 (PMC10817667; doi:10.3390/jpm14010054)
Supplement: Supplementary file 1 [file jpm-14-00054-s001.zip › jpm-2731018-supplementary.pdf]

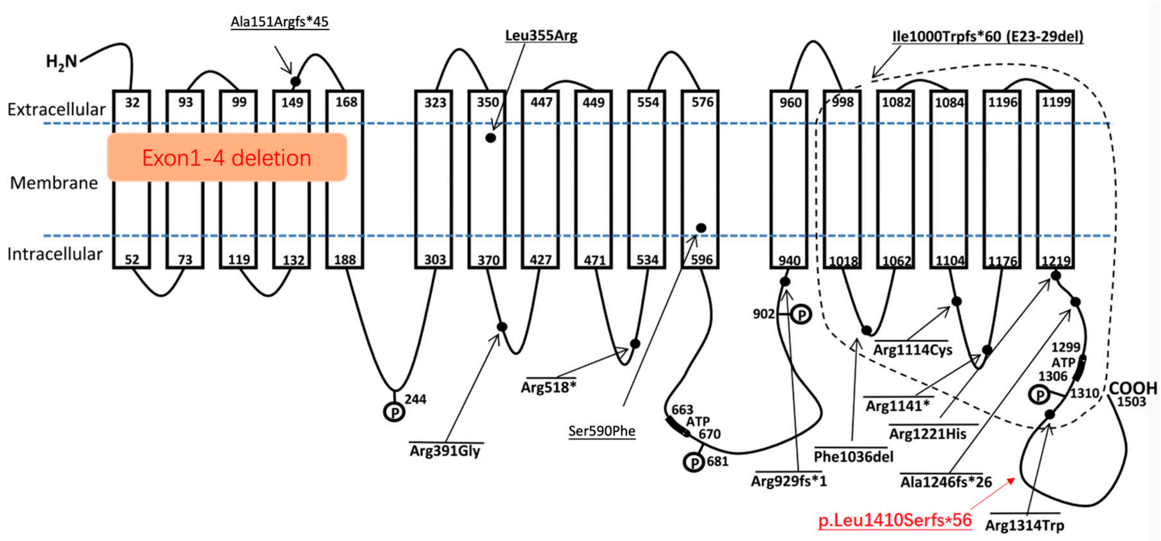

Figure S1 Summary of *ABCC6* gene mutations that cause GACI disease, including this study (red text) and literature (black text)
